# Estimating the Probability of a Major Outbreak from the Timing of Early Cases: An Indeterminate Problem?

**DOI:** 10.1371/journal.pone.0057878

**Published:** 2013-03-06

**Authors:** Meggan E. Craft, Hawthorne L. Beyer, Daniel T. Haydon

**Affiliations:** 1 Boyd Orr Centre for Population and Ecosystem Health, University of Glasgow, Glasgow, United Kingdom; 2 Department of Veterinary Population Medicine, University of Minnesota, St. Paul, Minnesota, United States of America; 3 ARC Centre of Excellence for Environmental Decisions, University of Queensland, Brisbane, Queensland, Australia; INSERM & Universite Pierre et Marie Curie, France

## Abstract

Conservation biologists, as well as veterinary and public health officials, would benefit greatly from being able to forecast whether outbreaks of infectious disease will be major. For values of the basic reproductive number (*R*_0_) between one and two, infectious disease outbreaks have a reasonable chance of either fading out at an early stage or, in the absence of intervention, spreading widely within the population. If it were possible to predict when fadeout was likely to occur, the need for costly precautionary control strategies could be minimized. However, the predictability of even simple epidemic processes remains largely unexplored. Here we conduct an examination of simulated data from the early stages of a fatal disease outbreak and explore how observable information might be useful for predicting major outbreaks. Specifically, would knowing the time of deaths for the first few cases allow us to predict whether an outbreak will be major? Using two approaches, trajectory matching and discriminant function analysis, we find that even in our best-case scenario (with accurate knowledge of epidemiological parameters, and precise times of death), it was not possible to reliably predict the outcome of a stochastic Susceptible-Exposed–Infectious-Recovered (SEIR) process.

## Introduction

If the basic reproductive number, *R*_0_, of an introduced pathogen does not greatly exceed one, outbreaks of infectious disease in small, closed populations may either be minor as a result of stochastic fadeout after a small number of transmissions, or major in which case fadeout only occurs after infection has spread through the majority of the population and only a few susceptible individuals remain [1], [2]. This bimodal nature of epidemic outcomes is of profound importance from a management perspective. If major outbreaks were predictable in the earliest stages of an outbreak, reactive control strategies could be implemented, such as vaccination, quarantine, or culling [3]–[6]. However, such measures are costly, time-consuming, inconvenient, and potentially reduce support for future interventions, thus it is not feasible to implement them for every outbreak. Despite its importance, the issue of being able to accurately predict whether outbreaks will be minor or major remains largely unexplored (but see [7]–[9]).

The distribution of final outbreak sizes is bimodal for values of *R*_0_>1 (though only weakly bimodal for values near 1), with the trough between peaks distinguishing minor from major outbreaks. Anderson & Watson (1980) provide an analytic solution for predicting the probability that an outbreak will be major (*p_m_*) for a SEIR (Susceptible-Exposed-Infectious-Removed) process with (a) gamma-distributed incubating (exposed) and infectious periods and (b) initial conditions that include various numbers of incubating or infectious individuals [10]. When exact initial conditions are known, and *R*_0_>1, stochastic simulations confirm that *p_m_* reliably predicts the probability of a major outbreak.

**Figure 1:**
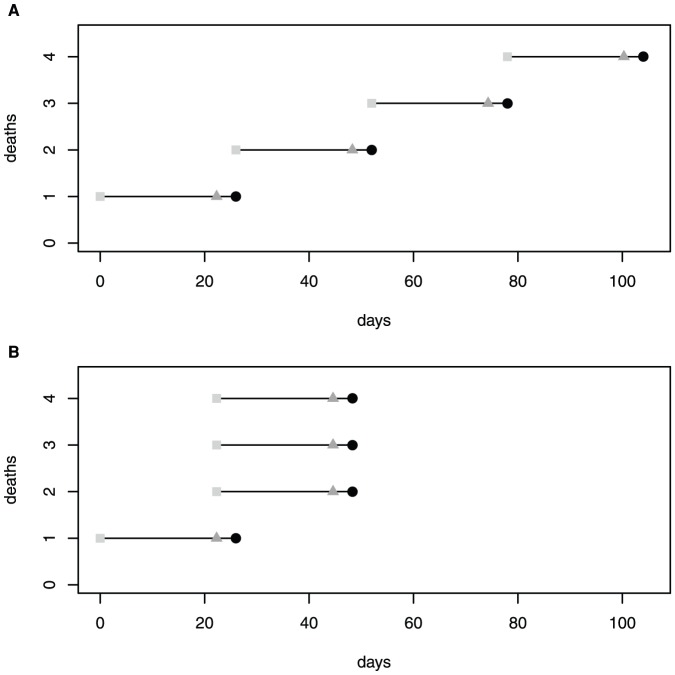
Outbreak schematic of our motivating assumption. Schematic of two infection histories of the first four infected individuals in an outbreak illustrating the times of infection (light gray squares), transitions from incubation to infectious period (gray triangles), and death of infectious individuals (black circles). We hypothesize that minor outbreaks are more likely when intervals between death times are wide and the number of concurrently infected individuals is low (A) and major outbreaks should occur with higher probability when death times are clustered and several individuals may be simultaneously infected (B). A more realistic pattern of deaths would include increased variability in incubating and infectious periods.

Several factors influence whether an epidemic will be major or minor, including: *R*_0_, the shape of the distribution describing the infectious period, and the numbers of incubating and infectious individuals at the time of prediction (i.e., the state of the system at the time of the *x* ^th^ death, denoted **θ** *_R = x_*). However, neither epidemiological parameters nor the timings of particular infection or removal events are likely to be known with certainty [11]. Even for closely observed epidemic processes, we rarely know the number of incubating individuals (E), we sometimes know the incidence of infected (I) individuals, and for fatal diseases we may have estimates for the number of dead (R) individuals. Intuitively, we expect that useful information for predicting final outbreak size may be contained in observable data: specifically the patterns of death times at the beginning of an outbreak (see Figure 1 for a hypothetical schematic). This study was originally motivated as a result of requests to advise on whether or not reactive vaccination programs should be initiated for the control of rabies outbreaks in Ethiopian Wolf (*Canis simensis*) populations. This prompted a more detailed examination of conventional infectious disease models. The objective of this paper is to consider whether the timing of early cases can be used to determine the likelihood of a minor or major outbreak.

We evaluate the ability of two estimation techniques, trajectory matching and discriminant function analysis (DFA), to predict whether the final size of outbreaks will be minor or major based on the timings of observed deaths in the early stages of the outbreak. We assume the best-case scenario where we have precise knowledge of the times of all deaths and the underlying epidemiological parameters, such as *R*_0_ and the shapes of the distributions describing the incubation and infectious periods. Even under these optimal conditions we find that these techniques provide very little predictive information regarding final outbreak size.

## Materials and Methods

The premise of our approach is to take as truth a simple epidemiological model that does not include demography (i.e. birth and natural death processes), assume that we know parameters precisely and that they do not vary, and then quantify our ability to predict whether an outbreak will be major or minor using two methods. This analytical approach was designed to maximize opportunities for finding predictive relationships between the timing of the first few deaths and outbreak size and we specifically explore conditions where both minor and major outbreaks are probable. Our initial intention to add increasingly complex forms of stochastic variation was redundant as, even under the best-case scenarios, essentially no predictive power was identified.

### (a) The epidemiological model

Stochasticity is often introduced into SEIR models through observational noise, process noise, and most commonly, event-driven approaches [12], [13]. Because dynamics at the beginning of infectious disease outbreaks inherently deal with small numbers of infected individuals, we focus on individual-based (event-driven) stochastic methods [14]. Here, we constructed a stochastic SEIR compartment model with no births or natural deaths [2] (Table 1). Dynamics were simulated using Gillespie's Direct Method [15], [16] with density-dependent transmission in a closed, well-mixed population. Such a model could be used to characterize many different diseases, but here, we have in mind the spread of a fatal disease (e.g. rabies [17]). Although the upper limit on the size of the population is not critical to our arguments, we examined disease dynamics in a population of 200 individuals (as might be representative of an endangered population of Ethiopian wolves) using two values of *R*_0_ that are consistent with rabies in canids (*R*_0_ = 1.2 [18]) and with a greater bimodal separation of minor and major outbreaks (*R*_0_ = 1.8). For each value of *R*_0_ we explored two scenarios (Table 2). For the first scenario, we assumed conventional exponential distributions for both the exposed and infectious period distributions (denoted 1∶1 EPD/IPD). However, because exponentially distributed transition times tend to overestimate the variance of the distribution, we also tested a second scenario with gamma distributed transition times, which may be more biologically realistic than the exponential. For the second scenario, we used the method of stages to limit the dispersion of exposed and infectious period distributions [19], [20] adopting three exponentially-distributed stages for both periods (denoted 3∶3 EPD/IPD). For each *R*_0_ value and for each EPD/IPD scenario (i.e. four models), we assumed a mean 22.3 day incubation period, a mean 3.7 day infectious period, and 100% fatality rate for infected individuals [18]. All simulations and analyses in this manuscript were performed in R [21].

**Table 1:** SEIR model with demographic stochasticity.

Parameters
*S* = number of susceptible individuals
*E* = number of exposed/incubating individuals
*I* = number of infectious individuals
*R* = number of removed/recovered/dead individuals
*N* = total population size where *S*+*E*+*I*+*R* = 200 individuals
σ = 1/incubation period, where the incubation period is 22.3 days
γ = 1/infectious period, where the infectious period is 3.7 days
*m* = the number of stages (compartments) used to model the exposed period, where *m* = 1 or 3
*n* = the number of stages (compartments) used to model the infectious period, where *n* = 1 or 3
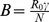 , where *R*_0_ = 1.2 or 1.8

**Table 2:** Study design for each model.

*R*_0_	Scenario	*m*	*n*	Resulting distribution	Notation
1.2	1	1	1	exponential	1.2, 1∶1
1.2	2	3	3	gamma	1.2, 3∶3
1.8	1	1	1	exponential	1.8, 1∶1
1.8	2	3	3	gamma	1.8, 3∶3

For each *R*_0_, two scenarios are tested which are described by the number of stages in the exposed period distribution (*m*) and the number of stages in the infectious period distribution (*n*). The abbreviated model notation is also included.

### (b) Simulating a wide range of final outbreak sizes

To explore whether the pattern of early deaths could predict the probability of a major outbreak, we used our SEIR model to simulate hypothetical outbreaks. To ensure we evaluated a wide range of outbreaks—in case final outbreak sizes produced by ‘rare’ outbreaks are more or less predictable than ‘common’ outbreaks—a stratified sample of outbreaks was generated based on the probability of a major outbreak, *p_m_*. Based on Anderson & Watson (1980), *p_m_* = 1- *π^ψ^* where *π* is the smaller root of:
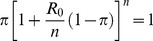
(1)and *n* is the number of stages used to represent the infectious period. The parameter *ψ* is a function of the initial conditions:
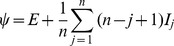
(2)where *E* is the total number of incubating individuals and *I_j_* is the number of infectious individuals at stage *j* [10]. The value *ψ* represents the weighted sum of exposed and infectious individuals; all incubating individuals are fully counted (valued at 1) because they have the entire infectious period yet to come, but in cases where there are multiple infectious period stages (*n*>1) it is necessary to discount the value of the individuals that are already in the later stages. The number of incubating period stages affects neither the value of *ψ*, nor *p_m_*; similarly the population size of susceptible individuals is not present in Equation 1, and thus cannot directly influence the analytical estimate of the proportion of outbreaks that would be major. Note that because *n*, *E* and *I* are integers, there are only a finite number of values that *p_m_* can be for a given value of *R*_0_, and *π* is a decreasing function of *n* such that the probability of a major outbreak increases as a function of *n*. In order to stratify our sampling efforts based on *p_m_*, we simulated an outbreak until the x^th^ death occurred; we then effectively ‘froze’ or stopped the simulated outbreak, and recorded the state of the system at that moment (i.e. the number of incubating and infectious individuals) and the epidemiological parameters (*R*_0_, *n*). These values were then used in Equations 1 and 2 to calculate *p_m_* deterministically. Stratification balanced sampling across a wide range of expected outcomes, thereby further maximizing opportunities for detecting predictive relationships.

All simulations were started with an identical initial condition (*E_t = _*_0_ = 1). In order to have a consistent number of death times to evaluate across all simulations, only outbreaks with at least *x* deaths were retained for inclusion in the analysis. Here we attempt to predict whether the outbreak will be major or minor at the time of the 4^th^ death (*x* = 4) as this corresponds closely to the real-world problem of having to decide whether to launch a control program to protect a population at threat at the beginning of a potentially major outbreak [17]. For each simulation, we computed and recorded (i) the times of the first four deaths, (*t*_1_, … , *t*_4_), (ii) the final size of the outbreak, which was used to classify the outbreak as minor or major (through use of the k-means clustering method with two centers [22]), and (iii) *p_m_* at the time of the fourth death. We ran simulations until we found outbreaks satisfying a wide range of *p_m_* values. Specifically, we searched for outbreaks with *p_m_* in each of 10 strata for intervals of 0.1 between 0 and 1. We evaluated two techniques (trajectory matching and DFA) for predicting the final outbreak size of these stratified “observed” outbreaks. For each of the 10 *p_m_* strata, we searched for 20 outbreaks for the trajectory matching technique and for the DFA we simulated 5000 outbreaks, or until 1 million simulations had been scanned per interval.

### (c) Trajectory matching

The premise of trajectory matching is to find simulated outbreaks that match characteristics of a single observed outbreak to within a defined tolerance (in our case these characteristics were the timings of the first four deaths). The matching simulations are then forward simulated to determine the number of deaths of the matching outbreak, thereby developing an empirical frequency distribution of outcomes, and where each outbreak can be classified as major or minor. If trajectory matching were a useful technique we would expect to find that matched trajectories tend to predict the true outcome better than at random.

We simulated 20 *observed_tm_* outbreaks in each of the 10 *p_m_* strata. Thus, there could be a maximum of 200 *observed_tm_* outbreaks for each of the 4 models examined (Table 2) although, as expected, there were very few low values of *p_m_* at higher values of *R*_0_ (*R*_0_ = 1.8). For each *observed_tm_* outbreak, we then simulated 200 “matched” outbreaks where the timing of the first *x* deaths was similar to the *observed_tm_* outbreak (or until 1 million simulations were scanned). A simulated outbreak was deemed similar enough, or ‘matched’, if:
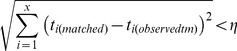
(3)where *η* is a tolerance value to be chosen and *t_i(matched)_* is the *i* ^th^ death time from the matched outbreaks while *t_i(observedtm)_* is the *i* ^th^ death time from the *observed_tm_* outbreaks. Equation 3 is a generalization of the Pythagorean theorem in Euclidean *x*-space. A large value of *η* represents very inclusive criteria, whereas a small value of *η* would select for a narrower range of outbreaks where the timings of deaths are very similar. There is a tradeoff when selecting a value for *η*. For example, setting *η* to a small value would select for simulations with more similar death times, however this becomes more computationally intensive as more simulations must be scanned in order to find these ‘good fits’. Although the specific value of *η* is not crucial to our argument, here we show results for *η* = 4. This value of *η* would intuitively correspond to a scenario where each of the four *t_i(matched)_* could be different from *t_i(observedtm)_* by up to two days. In the electronic supplementary information we also show results for a sensitivity analysis at more ‘narrow’ matching criteria of *η* = 2 and 3. For each unique value of *p_m_* from *observed_tm_*, we pooled the corresponding *matched* outbreaks and calculated the mean proportion of predicted major outbreaks. We then evaluated the predictive power of trajectory matching by comparing the proportion of major outbreaks among the 200 *matched* outbreaks to the calculated value of *p_m_* (at the time of the *x* ^th^ death) for the corresponding *observed_tm_* outbreak.

### (d) Discriminant function analysis

Discriminant function analysis (DFA) can be used as a classification tool [23]. DFA can quantify how well known explanatory variables contribute to correct classification of known categorical response variables. The end result is a model where the explanatory variables predict the group classification; the models can have poor or good predictive power. For the purposes of this manuscript, we used DFA to classify outbreaks as minor or major based on the time intervals between sequential deaths. We simulated 5000 outbreaks in each of the 0.1 intervals of *p_m_*, or until 1 million simulations had been run per interval (some values of *p*_m_ are rare for a given value of *R*_0_ resulting in fewer samples in these bins). These *observed_dfa_* simulations were used to evaluate whether quadratic DFA (which is a type of DFA that does not assume that the covariance matrix is identical for different classes) [23] could predict whether an outbreak would be minor or major based on the *x*-1 time intervals between the *i^th^* and *i^th^*-1 deaths (*i* = 2, 3, … , *x*). The explanatory variables for our discriminant function were the three time intervals between the four deaths, while the categorical response variable was whether an outbreak was minor or major. We used the qda function in the R package MASS [23] to construct a discriminant function on a random sample of half of the *observed_dfa_* outbreaks. We then used the discriminant function model to predict the classification of minor and major outbreaks for the remaining half of the *observed_dfa_* outbreaks. We compared the percentage of actual (*observed_dfa_*) minor and major outbreaks with the percentage of DFA-predicted minor and major outbreaks. We used the kappa statistic 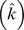 to measure agreement, where <0 is poor, 0–0.2 is slight, and 1 is perfect prediction [24].

## Results

### (a) Simulated outbreak sizes

**Figure 2:**
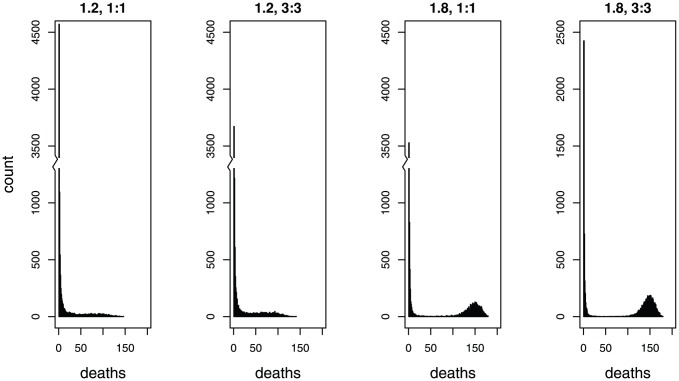
Outbreak sizes. Frequency distribution of outbreak sizes based on 10,000 stochastic simulations of four SEIR models with *R*_0_ of 1.2 or 1.8, and either exponentially distributed or gamma-distributed incubation and infectious periods (1∶1 and 3∶3, respectively). All outbreaks were retained for this figure regardless of the number of deaths.

For all 4 models (Table 2), outbreaks were characterized by final outbreak sizes ranging from 0–73% of the population when *R*_0_ = 1.2, and from 0–91% of the population when *R*_0_ = 1.8 (Figure 2). Outbreak size distributions are only weakly bimodal when *R*_0_ = 1.2, but are clearly separated when *R*_0_ = 1.8. Predictive power is expected to be highest when the outbreak size distributions do not overlap, and slightly reduced when a degree of overlap in the size distributions impairs our ability to classify an outbreak as major or minor. Overall, we found that both estimation methods performed poorly at predicting the outcome of an epidemic based on the timing of the first four deaths.

### (b) Trajectory matching

**Figure 3:**
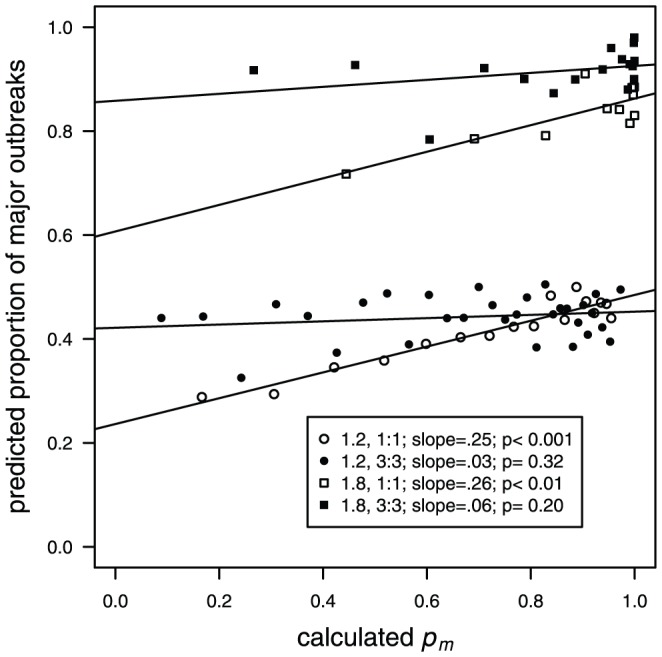
“Matched” versus “calculated” probabilities. The proportion of estimated *matched* outbreaks that are major (y axis) compared to calculated *p_m_* values from the corresponding *observed_tm_* outbreaks at the time of the 4^th^ death (x axis) for each of the four models. Each ‘predicted proportion of major outbreaks’ point on the figure represents the average of 200 to 4000 *matched* outbreaks. The lines represent fitted values from a linear regression model.

If the predicted proportions of major outbreaks from matched simulations and calculated values of *p_m_* were similar, this would show that information contained in the times of the first few deaths could be used to reliably predict the probability of a major outbreak. For perfect agreement, we would expect a slope of one on a linear regression between *p_m_* calculated using Equations 1 and 2 for each of the *observed_tm_* outbreaks and the predicted proportion of major *matched* outbreaks per *p_m_* value. Although there was a positive relationship between calculated *p_m_* and the predicted proportion of major outbreaks, (statistically significant for both 1∶1 EPD/IPD scenarios), the slopes of the linear regressions were far below the expected unit value, as the highest slopes were only 0.25 and 0.26 for the 1∶1 EPD/IPD scenarios, and close to 0 for the 3∶3 EPD/IPD scenarios (Figure 3). Even though the *observed_tm_* outbreaks were selected to vary across the fullest possible range of values of *p_m_*, the proportion of major outbreaks in the *matched* simulations only varied from 0.28 to 0.50 for *R*_0_ = 1.2 and from 0.72 to 0.98 for *R*_0_ = 1.8. This result did not change with our sensitivity analysis for lower values of *η* = 2 and 3 (Figure S1).

### (c) Discriminant function analysis

When *R*_0_ = 1.2, 47% of the 1∶1 and 40% of the 3∶3 scenarios for the *observed_dfa_* outbreaks were major; when *R*_0_ = 1.8, 70% and 63% of *observed_dfa_* outbreaks were major (1∶1 and 3∶3, respectively). If our DFA method were perfect in predicting outbreak size, we would expect 100% agreement between actual *observed_dfa_* major and DFA-classified major outbreaks, and 100% agreement between minor outbreaks. For *R*_0_ = 1.2, 1∶1 and both *R*_0_ = 1.8 scenarios, DFA correctly classified major outbreaks quite well (Table 3). However, because DFA classified almost everything as major for these models, DFA performed poorly in predicting minor outbreaks. For *R*_0_ = 1.2, 3∶3, DFA predicted minor outbreaks slightly better than random, and was poor at predicting major outbreaks. Overall the agreement between actual outbreak size and DFA-predicted outbreak size was no better than by chance (−0.009<
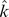
<0.007).

**Table 3:** Percentage of major or minor outbreaks classified by DFA as minor or major and the kappa statistic for each model.

1.2, 1∶1, kappa = 0.003	DFA classified as minor	DFA classified as major
Actual observed*_dfa_* minor	22.5	77.5
Actual observed*_dfa_* major	22.2	77.8
1.2, 3∶3, kappa = −0.009	DFA classified as minor	DFA classified as major
Actual observed*_dfa_* minor	62.5	37.5
Actual observed*_dfa_* major	63.3	36.7
1.8, 1∶1, kappa = 0.002	DFA classified as minor	DFA classified as major
Actual observed*_dfa_* minor	11.7	88.3
Actual observed*_dfa_* major	11.6	88.4
1.8, 3∶3, kappa = 0.007	DFA classified as minor	DFA classified as major
Actual observed*_dfa_* minor	5.8	94.2
Actual observed*_dfa_* major	5.3	94.7

Trajectory matching likely performs better than DFA because it captures more of the unobserved process (the number of incubating individuals) as compared to DFA, which is solely based on time intervals between deaths.

## Discussion

The probability that an outbreak in our *observed* samples will be major can vary from <0.1 to >0.9 (e.g. Figure 3) depending on the fully specified condition at the time of the *x* ^th^ death (**θ** *_R = x_*). While this information could be valuable in deciding whether intervention measures in the early stages of an infectious disease outbreak are required, the number of incubating and infectious individuals is likely unknown, hence the probability of a major outbreak is not directly estimable using Equations 1 & 2 alone. Instead, we tested whether information relating to the probability of a major outbreak might be recovered from directly observable data: namely the incidence of deaths. While intuitively we expect that early death times, or specifically the pattern of these death times, would help to predict final outbreak size, our proposed techniques are unable to predict the outcomes of the SEIR processes we examine, even given a realistic and arguably highly optimistic knowledge of the epidemiological parameters and timings of deaths.

Once the number of observable deaths increases, major outbreaks are more probable. For example, the average probability of a minor outbreak given the occurrence of 5 deaths in our model was only 0.08 for *R*_0_ = 1.8, 3∶3, and this probability then drops to 0.06 for 6 observed deaths. For low values of *R*_0_ (1.2 and 1.8) predicting outbreak size appears to be hampered by insufficient information (specifically a limited number of deaths), but by delaying the prediction and awaiting more information (deaths), the result becomes a foregone conclusion (since more deaths means a major outbreak is underway). For higher values of *R*_0_, there are few, if any fadeouts.

One contributory source of variation is clearly contained in the incubating and infectious period distributions. Although increasing the number of stages from 1 to 3 stages corresponds to an approximately three-fold reduction in the variance of the exposed and infectious period durations, the variance is still substantial. Second, this variance has a cumulative effect, so even small amounts of variation in the duration of the incubation and infectious periods can result in considerable variation in the timing of deaths after a few transmission events (e.g. by the fourth death). Third, there are other sources of variation that may also play an important role in eroding predictive power, such as the variation in the number of transmissions that results from each infectious case. For instance, even if there were no variation in the duration of the infectious period, when *R*_0_ = 1.2 the number of transmissions that occurs per infectious case is Poisson distributed with mean *R*_0_ and 95% confidence intervals of 0–4 transmissions. This is clearly also an important source of variability in transmission dynamics and could mask any potential gain in predictive power that might result from less variable exposed and infectious periods.

There are three issues that warrant further discussion. The first is that the trajectory matching approach might fail because our *matched* outbreaks are not similar enough and hence *η* must be smaller. However, our sensitivity analysis indicated prediction performance did not improve when *η* is reduced (Figure S1). We conjecture that because of the inherent variability characteristic of the early stages of a stochastic epidemic, a wide range of system states (**θ** *_R = x_*, and hence values of *ψ*) are likely consistent with any single temporal sequence of deaths, and that even reducing *η* to very low values will not help in procuring better predictors of *p_m_*. In other words, while there are certainly initial conditions that are much more likely to give rise to larger or smaller outbreaks than others (and Anderson and Watson's 1980 manuscript formalizes this relationship), the early time-of-death patterns generated from these initial conditions are also likely to be generated during outbreaks destined to be of many different final sizes. Therefore we cannot reverse the direction of inference and use time-of-death patterns to infer final outbreak size. This interpretation is consistent with the results of our DFA.

Second, we expected that the 3∶3 scenario would perform better than the 1∶1 scenario due to less dispersed incubating and infectious periods and more certainty around the means, yet the opposite was true (Figure 3), though it must be emphasized that neither scenario predicted well. We further note that some of our conclusions may be influenced by the relative durations and variances in the exposed and infectious periods, and this could be an avenue for future studies. Our case study for this manuscript was rabies, so this work was based on a model where the duration of the incubation period was approximately 7 times larger than the infectious period. It is known, however, that the ratio of the durations of these periods can affect dynamics [20], [25], and that some of the details regarding how predictability changes as a function of variation in the exposed and infectious periods may be influenced by the specifics of model parameterization.

Finally, in this study we fail to predict outbreak size even assuming the underlying parameters of the system are fixed and known. Including either real variation or measurement error in these parameter values is likely to erode what little predictive power we have described.

Where there is the possibility of either minor or major outbreaks in a closed, well-mixed stochastic SEIR setting, we come to the surprising conclusion that outcomes are essentially indeterminate given a realistic knowledge of the epidemiological process, and we therefore caution against possible over-interpretation of timings of early incidences. We note that the distribution of cases among groups may be more informative in structured populations and that other methods or early outbreak incidence metrics might predict major outbreaks with more accuracy. We often find ourselves ‘blaming the data’ for the inability to forecast final outbreak size. The results from our analyses suggest the problem may be more fundamental.

## Supporting Information

Figure S1 **“Matched” versus “calculated” probabilities and sensitivity analysis.** The mean proportion of estimated *matched* outbreaks that are major (y axis) compared to calculated *p_m_* values from the corresponding *observed_tm_* outbreaks at the time of the 4^th^ death (x axis) for *R*_0_ = 1.2 and 1∶1 EPD/IPD for *η* = 2, 3, and 4. The lines represent fitted values from a linear regression model. All *η* had p<0.001.
(DOC)
