# Supplementary material for: Estimating the Probability of a Major Outbreak from the Timing of Early Cases: An Indeterminate Problem?
Source: PLoS One. 2013 Mar 6;8(3):e57878. doi: 10.1371/journal.pone.0057878 (PMC3590282; doi:10.1371/journal.pone.0057878)
Supplement: Figure S1 — “Matched” versus “calculated” probabilities and sensitivity analysis. The mean proportion of estimated matched outbreaks that are major (y axis) compared to calculated pm values from the corresponding observedtm outbreaks at the time of the 4th death (x axis) for R 0 = 1.2 and 1∶1 EPD/IPD for η = 2, 3, and 4. The lines represent fitted values from a linear regression model. All η had p<0.001. (DOC) [file pone.0057878.s001.doc]

**
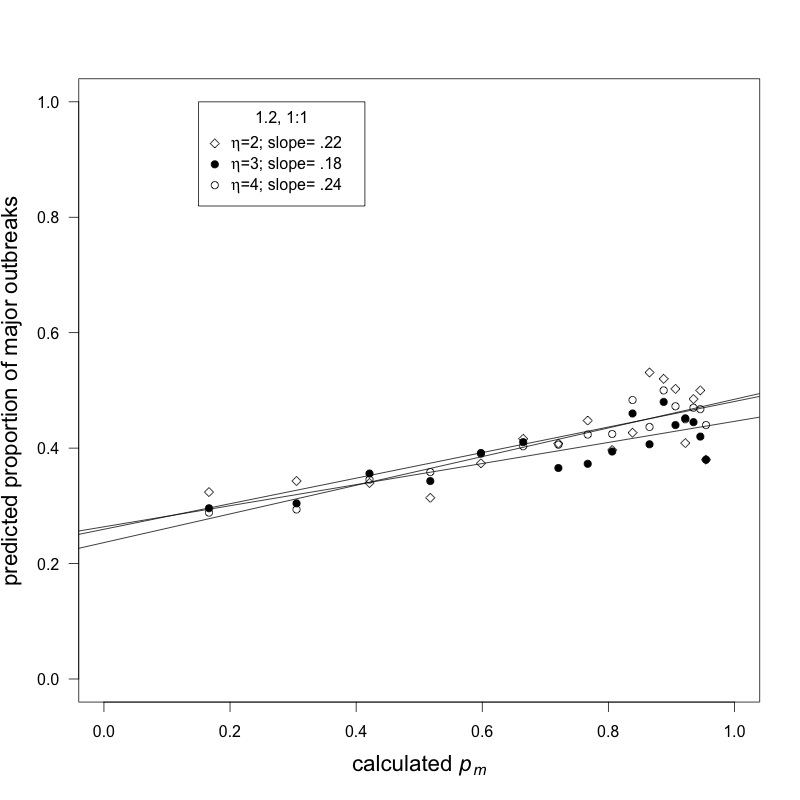
**

**Figure S1.** The mean proportion of estimated *matched* outbreaks that are major (y axis) compared to calculated *p_m_* values from the corresponding *observed_tm_* outbreaks at the time of the 4^th^ death (x axis) for *R*_0_=1.2 and 1:1 EPD/IPD for *η* = 2, 3, and 4. The lines represent fitted values from a linear regression model. All *η* had p < 0.001.
